# What are the Facilitators and Obstacles to Participation in Workplace Team Sport? A Qualitative Study

**DOI:** 10.3934/publichealth.2017.1.94

**Published:** 2017-02-23

**Authors:** Andrew Brinkley, Josie Freeman, Hilary McDermott, Fehmidah Munir

**Affiliations:** 1 School of Sport, Exercise and Health Sciences, National Centre for Sport and Exercise Medicine, Loughborough University, Loughborough, Leicestershire, United Kingdom, LE11 3TU; 2 Atos, Atos IT Services, Regents's Place, 4 Triton Square, London, United Kingdom, NW1 3HG

**Keywords:** barriers, enablers, organisational, physical activity, tailoring, template analysis

## Abstract

Working age adults are failing to meet physical activity recommendations. Inactive behaviours are increasing costs for diminished individual and organisational health. The workplace is a priority setting to promote physical activity, however there is a lack of evidence about why some employees choose to participate in novel workplace activities, such as team sport, whilst others do not. The aim of this study was to explore the complexity of facilitators and obstacles associated with participation in workplace team sport. Twenty-nine semi-structured face-to-face and telephone interviews were conducted with office workers (58% female) (36 ± 7.71) from manufacturing, public services, and educational services. Data was analysed through template analysis. Five sub-level (i.e., intrapersonal, interpersonal, organisational, community and societal influences) facilitate participation or create obstacles for participants. Participants were challenged by a lack of competence, self-efficacy, negative sporting ideals and amotivation. Unhealthy competition, an unstable work-life balance and unsupportive colleagues created obstacles to participation. An unsupportive organisation and workplace culture placed demands on workplace champions, funding, facilities and communication. Healthy competitions, high perceptions of competence and self-efficacy, and being motivated autonomously enabled participation. Further, relatedness and social support created a physical activity culture where flexible working was encouraged and team sport was promoted in accessible locations within the organisation. Researchers should consider accounting for complexity of these influences. A participatory approach may tailor interventions to individual organisations and the employees that work within them. Interventions whereby autonomy, competence and relatedness are supported are recommended. This may be achieved by adapting sports and training workplace champions.

## 1. Introduction

### 1.1. Background

Inactivity and sedentary behaviour are well-established modifiable risk factors for non-communicable illness and conditions, known to contribute to premature mortality [1]. These include coronary heart disease (CHD), type 2 diabetes, obesity, osteoporosis, some forms of cancer and diminished mental health outcomes (e.g., depression) [2],[3]. Recent estimations indicate the direct health care and indirect costs (e.g., productivity) for inactivity's impact on CHD, stroke, type 2 diabetes, breast cancer and colon cancer totalled $67.5 billion globally ($18.49 million direct health care costs in the UK) in 2013 [4].

Adults in high-income countries are struggling to reach and maintain minimum national and international physical activity guidelines (i.e., 150 minutes of moderate or 75 minutes of vigorous physical activity per week) [1],[5],[6]. For example, in 2012 only 67% of working age men and 55% of working age women reported meeting minimum weekly physical activity guidelines in the UK [6]. Therefore, improving participation in physical activity remains a priority of public health policy [1]–[4].

### 1.2. The Workplace and Physical Activity

An employee's physical activity behaviour is associated with the health of the organisation [4],[7],[8]. Indeed, sickness absenteeism and sickness presenteesim are known to be influenced by the adoption of inactive and sedentary behaviours [9],[10]. Likewise, empirical evidence has associated antecedents of poor work performance (e.g., low productivity; fatigue; work engagement) and employee turnover (e.g., low job satisfaction; high job stress; muscular-skeletal pain; burnout) with an inactive workplace [11]–[14]. Evidence indicates these factors attributable to inactivity alone contributed to an observed $13.7 billion global loss in productivity in 2013 [4]. Further, sedentary working environments (e.g., deskbound roles) and inactive working behaviours may contribute to the presence and costs of non-communicable illnesses, injuries and conditions [2],[15]. Therefore, promoting workplace physical activity remains a priority for occupational health promotion [4],[16],[17].

The workplace provides opportunities, funding streams and support for the promotion of physical activity, whilst the workforce is a stable population with a consistent level of exposure to physical activity programmes [7],[8],[18]. The complexity of participation should however be considered. Organisational factors such as the accessibility of facilities [19]; presence of funding [20]; and available time may influence participation in workplace physical activity [21]. Further, psychosocial factors such as the attitudes of colleagues, superiors and workplace champions (i.e., an employee adopting ownership and responsibility for delivering health promoting opportunities to their colleagues), and support from within the workplace culture may encourage or discourage participation [22],[23].

### 1.3. Participation in Workplace Team Sport

A recent review has demonstrated the benefits of team sport within a workplace setting [24]. Evidence from experimental designs indicates participation in workplace team sport has the capacity to improve individual health outcomes (e.g., cardiorespiratory health, musculoskeletal function, psychological well-being) and organisational health outcomes (e.g., productivity, sickness absence, workability) in a similar degree to workplace physical activity interventions (e.g., walking, active transport) [25]–[28]. Further, participation in workplace team sport may have additional social benefits, which are yet to be identified through workplace physical activity programmes. These include, positively influenced relationships, communication and team cohesion within the workplace [29]–[34].

However, poorly described homogeneous samples and a lack of empirical evidence limits the research examining workplace team sport [24]. Critically, there is a lack of qualitative evidence exploring why employees participate in workplace team sport and the facilitators and obstacles these individuals encounter. While extensive evidence discussing the enablers and barriers faced by children, young adults and the elderly is available in a sporting context, the same cannot be said for working age adults [35]. Certainly, this gap in research limits the effectiveness and sustainability of future team sport programmes.

To date, six qualitative studies have explored the facilitators and obstacles associated with participation in workplace team sport [34]–[39]. While this evidence offers some insight, it should be noted these studies broadly investigated participation in workplace physical activity rather than team sport directly, and therefore the findings are open to interpretation.

For example, low perceptions of self-competence have created obstacles for employees considering playing football with their colleagues [35]. While the evidence evaluating “Workplace Challenge” (i.e., a workplace health promotion scheme focusing on team sport) indicates the attitudes of employees and workplace champions can influence the workforces' participation in team sport [37]–[39]. Likewise, a study of workplace wellness schemes (some of which contained workplace team sports) suggests participation is influenced by the culture within the workplace [36]. Finally, the management and communication of opportunities may create acceptance within peer-groups [34].

For the promotion of workplace team sport to be successful, the specific obstacles and facilitators must be considered. A comprehensive understanding of these factors may allow researchers and practitioners to successfully tailor team sport into a workplace setting.

### 1.4. An Ecological Perspective

Evidence suggests physical activity behaviour in complex activities such as workplace team sport can be best understood through an ecological approach [40]. An ecological approach suggests the behaviour underpinning participation is influenced by intrapersonal, interpersonal, organisational, environmental and societal determinants [40].

*Intrapersonal* determinants reflect how psychological and biological factors enable or challenge behaviour [41]. A psychological influence may be perceptions of competence, while a biological influence may be a chronic health condition [42]. *Interpersonal* determinants refer to socially desirable factors. For example, employees are known to seek the support of colleagues or conform to the attitudes of managers [34]. *Organisational* determinants are influences on a workplace, departmental or cultural level [40]. For example, individual behaviour may be influenced by the working practises of an organisation [22]. *Environmental* determinants reflect logistics and structural factors that influence behaviour. For example, facilities may encourage or discourage participation in workplace team sport [37],[38]. *Societal* determinants refer to how policy and society driven attitudes (e.g., gender inequality) influence individual behaviour and experiences and perceptions of sport [40].

Participation in workplace team sport is a complex process that may be influenced from a societal, organisational, social and psychological standpoint [34]–[39]. Therefore, this study adopted an ecological approach to understand the facilitators and obstacles reported by participants [40]. The primary aim of this exploratory study was to gain an understanding on what determinants facilitate and challenge participation in workplace team sport.

## 2. Methods

### 2.1. Research Design

An exploratory design using semi-structured face-to-face and telephone interviews explored what facilitates participation and what creates obstacles for participation in workplace team sport [43]. The trustworthiness of face-to-face interviews is well established [43], while telephone interviews allow the researcher to collect data around the demands balanced by participants [44]. Ethical approval was granted from Loughborough University's Human Participants sub-committee.

### 2.2. Sampling

Purposive sampling was used to recruit employees participating and not participating in workplace team sport. Participants were recruited from private and public organisations in the UK (i.e., manufacturing and sales; public services; and educational services) [45]. A representative from occupational health was contacted by email and/or telephone to explain the purpose of the study and to arrange data collection.

To insure data discussing a variety of experiences regarding workplace team sport was collected; all employees, line managers and workplace champions were sampled. Participants were excluded from participation if they were not permanently contracted members of staff. Participants were invited to take part in an interview by telephone or email by the researcher or by an occupational health representative within their organisation.

### 2.3. Procedure

An interview schedule guided data collection (see Table 1). This explored, (i) physical activity participation; (ii) workplace physical activity and team sport motivation; (iii) workplace team sport participation; (iv) workplace team sport facilitators and obstacles; (v) set-up, maintenance and adherence to workplace team sport; and (vi) closing statements.

From January 2015 to August 2015, twenty-four face-to-face individual interviews and five telephone interviews were conducted during working hours in café's, offices, meeting spaces and conference rooms by two of the researchers^1^ trained in interview techniques (i.e., active listening, encouraging discourse, generating rapport).

With the knowledge and consent of participants, interviews were recorded on a digital voice recorder (Olympus VN-7700). Open-ended questions were asked to encourage discourse while probes were used to encourage participants to expand on interesting responses. Interviews lasted between 30 and 50 minutes (*M* = 36 minutes).

### 2.4. Analysis

Recorded interviews were transcribed verbatim and a template analysis was undertaken to identify and define key themes within the data [46]. Template analysis has provided trustworthiness and reflexivity in previous occupational health research [46]. Template analysis uses priori-themes to guide analysis towards a given research question.

The initial priori themes were based on the ecological model [40], while familiarisation in the data created a series of preliminary codes. These codes were attached to the initial priori themes where appropriate. If a code could not be attached, a new theme was developed. Once completed, a template was produced. The themes were grouped into first (e.g., factors that facilitate taking part in workplace team sport); second (e.g., interpersonal factors influencing participation in workplace team sport); third (e.g., social approval, understanding and support); and fourth (e.g., shared experience and group membership) level themes. The template was revised until it reflected the complete data set. All members of the research team gave their consensus on the data by reviewing the identified themes.

## 3. Findings

### 3.1. Participant Demographics

Twenty-nine employees with a range of job roles took part in this study (72% in a position of superiority over their colleagues). These participants (58% female) were aged between 22 and 57 (36 ± 7.71), had worked at their organisation from 2 months to 28 years (6 ± 6.12) and worked within teams. All the participants sampled reported being in a good state of health and participated in physical activity in their leisure time or workplace (e.g., soccer, exercise classes, yoga). All the participants in this study worked in office based roles. Additional participant demographics are available in Table 2.

### 3.2. Context of the Study

Fifty five percent of the sample participated in office-based team sports, traditional team sports and individual-team sports. Of these sixteen employees, six women participated in workplace team sport. Office-based team sports can be conceptualised as team sports that take place in an office space or breakout area. These sports were organised by groups of employees and often the organisation provided equipment to participate (e.g., a table tennis table). Office-based sports were played during breaks in the day, and included *“badminton and table tennis [P14]”*. Further, participants reported participating in traditional team sports with colleagues such as “*basketball [P5]”, “ultimate frisbee [P1]”, “touch-rugby [P18]”, “softball [P19]”, “indoor/beach volleyball [P1]”, “squash [P2]”, “soccer [P29]”,* and *“rounders [P22]”*. These sports were encouraged, communicated and promoted by the organisation, however not directly funded. These opportunities were therefore self-funded and organised by individual employees passionate about sport. Traditional team sports took place offsite outside during lunchtime or after-working hours as part of stand-alone workplace events, tournaments or sports programmes. Finally, individual-team sports were participated in. These can be defined as individual sports with a competitive team goal such as “*swimming [P28]”* and *“cycling [P19]”*. The organisation funded and organised these activities for their employees. Individual-team sports took place offsite at funded facilities (e.g., local sports centres) during breaks in the working day or after-working hours.

Participants playing team sport with their colleagues reported the facilitators and obstacles they encountered (i.e., denoted with TS in quotations). In contrast, participants not playing team sport (i.e., denoted with NP in quotations) typically described their barriers to participation. However, in some cases these participants were considering playing workplace team sport, and therefore discussed what motivated them to contemplate participation.

### 3.3. Overview of Themes

The facilitators and obstacles underpinning participation in workplace team sport are represented by the ecological model [40], and its intrapersonal, interpersonal, organisational, environmental and societal themes (see Table 3). These themes emerged across all participants' data regardless of job role, superiority or the industry they worked within. The findings representing these themes are presented below.

### 3.4. Intrapersonal Factors Influencing Participation in Workplace Team Sport

#### 3.4.1. Motivated by Self-interest

Intrinsic factors could autonomously motivate participation in workplace team sport. Key factors for participation included a preference for the type of team sport offered (e.g., *“I do it because I like the activity [P1, female health promotion manager aged 34, TS]”*) and feelings of enjoyment (e.g., *“I like that volleyball is quite novel, I like that it's quite fun [P6, female researcher aged 24, TS]”*) when playing team sport. It appeared the satisfaction participants associated with “sport” could motivate them to play team sports in their workplace from their own free will (e.g., *“I love most sports to be honest. My love of sport gets me there [P2: female researcher aged 28, TS]”*).

In contrast, having no interest or connection with the team sport offered within the workplace could create amotivation for workplace team sport and an obstacle to participation:

“Yeah it's not going to be enjoyable enough to do purely for the sake of enjoyment. So, it's not something I want to do on a regular basis” [P4: female, aged 48, NP].

#### 3.4.2. Motivated by External Sources

Competition and incentives were frequently reported to positively influence participation in workplace team sport. Competitions typically took place between departmental teams, while incentives were offered to employees who were playing team sport. Rewards and competition created controlled motivation to participate:

“There is awards and stuff like that. Again, you make it a little more competitive to try and get more people involved because there's got to be a carrot at the end of it” [P12: female personal assistant aged 42, TS].

Alternatively, the behaviours associated with unhealthy competition (e.g., aggression, criticism and banter) could create obstacles and demotivate employees from participating (e.g., *“People get very competitive, like you did that wrong. That direct criticism, I don't like that” [P10: female ledger controller aged 34, NP]*).

#### 3.4.3. Perceptions of Perceived Competence and Self-efficacy

A salient factor reported by participants in this study was perceived competence, and its influence on self-efficacy. Low-efficacy was attributed to diminished perceptions of competence and this created an obstacle to participation. While positive perceptions of competence were linked with high self-efficacy and this created a facilitator to participation. Undesirable comparisons with a colleague's ability were described to reduce the perceptions of competence and self-efficacy of participants considering participating in team sport:

“When you get something like football you start thinking I wonder how good everybody else is and that just puts a bit more worry about joining in [P23: female personal assistant aged 42, NP]”.

Likewise, a fear of social judgements and a challenging experience of team sport may diminish perceptions of competence, self-efficacy and create an obstacle for regular participation in team sport:

“I have never been very confident at competitive sport, which probably pins down why I feel pressure from others. You don't want to let your team members down [P26: female marketing employee aged 28, NP]”.

However, low perceptions of competence and self-efficacy could be positively regulated by tailoring the rules of the sport and the style of play to the employees participating. In many cases, a workplace champion prompted this change and created a facilitator to participation:

“So they [the workplace champions] took the basic rules, rather than some of the intricacies, which stop the game flowing and they ignored some of the minor infringements, so it was just the major infringements that got called up. It made it a more enjoyable game and a more flexible game, but it still had the feel of the sport. It made things easier to achieve and understand. An easy game to play and a more enjoyable game ultimately” [P18: male sports development manager aged 41, TS].

Removing the rules and structure traditionally recognisable with team sports was reported to improve perceptions of competence, positively regulate self-efficacy and create a facilitator to participation. Likewise, sports that had not been played for some time were reported to have similar positive influence on perceptions of competence and self-efficacy:

“What we found was the sports that no one had played before or the least amount of people had played before, that had the biggest enjoyment factor. I think people felt equal going into it. It was new to everybody, so everyone was one the same starting point. Potentially team sports which people haven't experienced at school” [P18: male sports development manager aged 41, TS].

Sports that were not regularly played were not associated with the same diminished perceptions of competence which are perhaps related with regularly offered team sports. Further, the novelty of these sports may create more equal perceptions of competence and therefore facilitate participation within the workplace:

“With rounders you get people that you wouldn't normally pick up doing something traditionally sporty, say a football match or whatever, because I think rounders you pick up people because they are like rounders is not a proper sport, it's more of a game and they're like if I come along and I'm rubbish it doesn't matter, whereas with football they think if I come along I'm going to be the worst, and it's going to be embarrassing. The advantage of rounders is that it gets people who might not get involved because of competence reasons” [P22: male project manager aged 46, TS].

Finally, female participants contemplating playing team sport reported challenges relating to body image. For example, body image consciousness and social comparisons may reduce self-efficacy and create an obstacle to participation:

“It's a body image thing. Exercising in a group, it can be quite intimidating. Getting sweaty in front of other people. Then it's a vicious cycle then because you really want to lose weight” [P8: female team coordinator aged 27, NP].

### 3.5. Interpersonal Factors Influencing Participation in Workplace Team Sport

#### 3.5.1. Support from Colleagues and Managers

The attitudes and behaviour of colleagues and managers provided support for team sport within the workplace. For example, some participants supported their colleagues through the psychosocial (e.g., lack of self-efficacy) and organisational (e.g., job demands and expectations) obstacles associated with participation:

“People are more comfortable playing with their peers than playing on their own for the first time. It's perhaps easier. I think they're more likely to be active if their playing alongside people they know like their colleagues” [P18: male sports development manager aged 41, TS].

#### 3.5.2. Group Involvement, Cohesion and Relatedness

Participation in workplace team sport created social relationships and friendships within the workplace. The appeal of developing social relationships and the membership of a social group motivated some employees to participate in workplace team sport:

“If I were play sport for work then that would be for social reasons. So, I think the main reason you would have like organised team sport in the workplace would be for the social interaction side of it” [P15: male IT support manager aged 38, NP].

The attendance and support of these social groups provided relatedness and motivated participants to regularly participate:

“I loved doing it as a team because it encourages you. When we were finishing here, and it's been a busy day I would have been tempted to say, you know what, I won't do it tonight. With the team, it's like let's do this” [P9: female solicitor aged 34, TS].

#### 3.5.3. Family, Work-life Balance and the Influence on Perceptions of Available Time

The family, social and workplace commitments balanced by participants influenced the time available for participation in workplace team sport. Prioritising workplace demands and personal commitments created an obstacle and a lack of motivation for workplace team sport where participants viewed their participation as extra time at the workplace:

“If I'm going to spend the time on an activity outside work, I would rather spend it outside with family or where I am with friends. I am spending enough time at work already” [P4: female, aged 48, NP].

However, a facilitator could be created when these time-based challenges were managed organisationally through a consistent programme of team sport events and interpersonally through scheduling of the working day:

“As long as it's regularly in the calendar, then people can normally change their schedules. It's when it hops between days and times where I think it becomes hard” [P5: female academic aged 36, TS].

It was noted that participation during the day offered a form of participation that enabled most members of staff to participate in workplace team sport:

“So if it was incorporated into the working day. So, having like inter-centre competitions and stuff like that. That would be quite good for everyone” [P16: female business improvement manager aged 36, NP].

Participation during lunch-hours was challenged by social comparisons made with the behaviours of colleagues and superiors. Maintaining a professional image and the time taken to return to work created obstacles such as job stress and a loss of productivity for some participants:

“The hour for the sport, plus showering and everything thing else afterward. That's a barrier for me. I have to make time up for it” [P5: female researcher aged 36, TS].

Though, in some cases addressing these obstacles led participants to play team sport with their colleagues outside of working hours:

“We played at the end of the day so earliest we do it is say four o'clock. So, most people are done, so we're not getting in the way of work. To do something that actually becomes quite physical and you're going to get sweaty, and then you're going to need a shower and all of that. You hopefully more people because you're not going to have meetings and things like that” [P2: female researcher aged 28, TS].

However, this challenged participants with childcare commitments. Frequently the physical activity behaviour underpinning participation in workplace team sport could be challenged by the presence of children and associated responsibilities:

“Free time went when I had kids. I use to be able to nip off for forty-five minutes to go to the gym but now it's kind of finish work get home and get the kids their tea and play with them for a bit” [P23: male IT analyst aged 34, NP].

### 3.6. Organisational Factors Influencing Participation in Workplace Team Sport

#### 3.6.1. Support

Frequently, superiors were supportive of participation (e.g., *“yeah, our manager is very accepting of us wanting to do it” [P26: female marketer aged 28, NP]*). However, in some cases the attitudes of superiors (e.g., *“managers, they end up clock watching [P28, male manager aged 39, TS]”*) and the attitudes of colleagues (e.g., *“you're seen as not working by your peers [P5: female researcher aged 36, TS]”*) created obstacles to participation.

The demand employers place upon their workforce may indirectly influence the adoption of negative attitudes towards workplace team sport. For example, unsupportive attitudes from higher-tier management can discourage participation in workplace team sport:

“Senior management have commented about it not looking particularly good if people come into the building and there's people playing table tennis” [P21: female development manager aged 33, TS].

However, managers who understood the benefits of sport provided acceptance and motivation to participate, and support to adhere to these opportunities within the workplace:

“If you have got a leader who is fairly active, fairly sporty and kind of says I want to put this together and I'm going to be there, then more people will go. As they think, oh the boss is going, I'll go.” [P13: male, aged 42, TS].

Likewise, a duty of care for the health and wellbeing of the workforce provided support, acceptance, investment and a facilitator for participation in workplace team sport:

“You spend all of your time, all your day at work, it's part of your life experience. If they want you enjoy being at work and be productive and stay with the company then they should provide an environment that encourages that and funding and organising some sort of sports activity, is one way of creating a nice culture” [P22: male project manager aged 46, TS].

**Table 1.** Interview Schedule.

Content	Topics for discussion
**Physical Activity Participation**	Do you currently take part in physical activity? (Sport; exercise; occupational)
	How often do you take part? (Regularly, occasionally)
	Where do you take part? (Inside or outside work?) (With or without colleagues?)
	Perceptions of physical activity. (Do you do enough?)
**Physical Activity and Workplace Team Sport Motivation and Benefits**	What do you enjoy about physical activity?
	What motivates you to take part (facilitators) in physical activity/workplace team sport?
	What restricts your involvement (obstacles) physical activity/workplace team sport?
	How does physical activity benefit you?
	How does physical activity/workplace team sport benefit your working life?
	What can your company gain from having physically activity employees?
**Workplace Team Sport Participation**	Do you take part in workplace team sport?
	What are your thoughts on workplace team sport?
	Better or worse than physical activity? (prefer physical activity on your own?)
	Would you want to participate with your colleagues?
**Workplace Team Sport Benefits**	What benefits does or could workplace team sport hold for you (individual), your team (group) and workplace (organisation)?
**Workplace Team Sport Facilitators and Obstacles**	What would motivate you to attend? (facilitators)
	What would stop you attending (obstacles)
	Do you think there would be any workplace enablers or barriers associated with workplace team sport? (culture; bureaucratical; external; environments; facilities; funding; time; resources)
	What times would work best?
	What sports would you like?
**Set-up, Maintenance and Adherence**	How should workplace team sport be set-up, maintained and managed? (HR; individual; committee)?
**Closing Statements**	Overall do you think workplace team sport would hold positive/negative health benefits for the company or a worthwhile venture?
	Do you have any further thoughts on the idea or anything else to add?

**Table 2.** Participant Demographics.

Organisation (Industry)	Workplace Team Sport organisation and support	Number of Participants	Gender	Age	Qualifications Held	Marital Status	Department	Business Size	Job Role	Work in a Team/Numbers/In a Position of Superiority or Management	Contract Type	Tenure (Months–Years)
Manufacturing	Cycling and squash encouraged by the organisation, but self organised. Facilities offsite. Participation outside of working hours. Information not provided if activity was funded or self-funded.	10	6 Females (60%)	27–43 (37.1 ± 4.93)	Not Provided	Not Provided	HR (10%), Operations (10%), Legal (20%), Retail (20%), Group Development/Communication (10%), Public Relations (10%), IT (10%), Admin (10%)	Large	Manager (40%), Coordinator (20%), Solicitor (10%), Head of Department (20%), Personal Assistant (10%)	1/3 to 23 (*M = 9)/*90% in a position of superiority or management	All Full-time	18 Months–11 Years 6 Months (5.32 ± 3.5)
Manufacturing 2	Participants self-funded and organised soccer offsite outside of working hours. Swimming was funded, organised, supported and participated in during working hours at a facility offsite.	7	3 Females (43%)	27–57 (37.4 ± 10.17)	Further Education (14%) Degree (43%), Higher Degree (43%)	Single (43%), Married (47%)	Retail (14%), IT (14%), Design (28%), Product Development (14%), Ecommerce (14%), Marketing (14%)	Large	Manager (43%), Analyst (14%), Marketer (29%), Product Developer (14%)	1 to 6/3 to 16 (*M* = 6.4)/56% in a position of superiority or management	All Full-time	15 Months–28 Years (9.6 ± 18.5)
Public Services	Workplace challenge encouraged by the organisation, funded externally, participated in outside of working hours. Soccer, softball, rock climbing and cycling self-funded by participants, participated in outside working hours. Table tennis provided by organisation, played during lunch hours. All facilities offsite.	6	3 Females (50%)	22–41 (34 ± 7.58)	Not Provided	Not Provided	Human Resources (16%), Health Promotion (16%), Development (50%), Corporate Communication (16%)	Small/Medium (50%) Large (50%)	Advisor (15%), Practitioner/Consultant (15%), Manger (40%), Officer (30%)	All work as part of teams/80% in a position of superiority or management	All Full-Time	11 Months–12 Years (5.9 ± 4.57)
Education (Higher)	Netball, badminton and squash was self-funded. Played outside of working hours. Seasonal sports competitions (e.g., soccer), played during working hours. Organised in workplace, self-funded participation. All facilities on site.	6	5 Females (83%)	24–48 years (35.6 ± 9.6)	Higher Degree (50%), PhD (50%)	Engaged (17%), Single (50%), Married (33%)	Sport, Exercise and Health Science	Large	Researcher/consultant (17%), Researcher (33%), Senior Lecturer (33%), Project Manager (17%)	1 to 4/2 to 22/66% in a position of superiority or management	All Full-time	2 Months–16 Years (4.03 ± 5.97)

**Table 3.** Workplace Team Sport Template Analysis.

Ecological factors	Sub-theme	Facilitators (enablers) to team sport (+)	Obstacles (barriers) to team sport (-)
**Intrapersonal factors**	Motivated by self-interest	Enjoyment	Amotivation
		Preference for type of team sport	Lack of enjoyment in team sports
	Motivated by external sources	Incentives to participate	Unhealthy competition
		Schemes with rewards	
		Positive competition	
	Competence and self-efficacy	High perceptions of competence	Low perceptions of competence
		High self-efficacy	Low perceptions of fitness
		Modified rule and adapted sports	Low self-efficacy
		Novelty of sports	Low perceptions of body image
	Psychosocial support from colleagues and managers	Acceptance and social support	Lack of social support
		Shared experiences and group membership	
	Group involvement, cohesion and relatedness	Group cohesion	
	Family, work-life balance and the influence on perceptions of available time	Functional work-life balance	Family, work-life balance and perceptions of no available time
		Time, scheduling, work-life balance and multiple options	Workplace commitments and demands and the job
			Time of sport not fitting in with work and lifestyle
**Organisational factors**	The level of support for team sport	Support of colleagues, managers and the organization	Lack of support from colleagues, managers and the organization
			Perceptions of not working
	The organisation and management of team sport	Sharing responsibilities	No clear organization or management
		The importance of champions	Time burdened and constrained workplace champions
		Committees and a shared voice	Informal organisation and in-groups
		Organisational ownership and support	
		Human resources and occupational health	
	Funding team sport	Organisational funding	Lack of funding
		Willingness to self-fund	The public sector and accountability
			Unwilling or unable to self-fund
	Communication of team sport	Tailoring communication style to the structure of the organisation	Informal groups and communication
		Modern communication and social media	Limitations of intranet
			Lack of two-way communication
	Workplace culture and team sport	A supportive workplace culture	A discouraging workplace culture
		A flexible working culture	A culture which promotes working non-stop
**Environmental factors**	Sports and changing facilities	Available sports and changing facilities	Inaccessible facilities
		Accessible sports facilities	Health and safety challenges
		Utilizing the natural environment surrounding the workplace	Logistical and pragmatic obstacles
		Acceptance for changing time and returning to work	Unavailable facilities
			Poor weather
	The support of external sporting organisations	The positive impact of external sporting organisations	
**Societal factors**	Bias and inequality in sport		Past experience of school/youth sport and bias
			Sporting demographic ideals, everyday sexism and bias

#### 3.6.2. Organising and Managing Team Sport

Team sport was delivered by workplace champions, sports committees or by the organisation. A structured method of organisation created an enabler for participation, while a lack of management, structure and organisation created obstacles for participants.

An enthusiastic and committed workplace champion motivated participants to play workplace team sport: *“I do it because I have a particularly supportive champion [P1: female health promotion manager aged 34, TS]”*. However, obstacles were created when the demands of employment challenged a workplace champion's effectiveness. For example, champions lacked the time, ability or resources to effectively manage workplace team sport:

“In reality I have to do it, I would basically have to turn up every week and collect the money, make the booking, organise and pick the team, and I'm not going to do it, I'm not, I don't want to do that basically, I don't want to commit myself. I don't even live near here, I work across the county, I'm not going to be here till eight o'clock, for them to play football, for me then to drive forty miles back home. Practically it's not going to happen. It's important to have a group of staff members which are willing to put the effort in to make it work” [P17: male workplace health advisor aged 40, NP].

Therefore, the pressure placed on champions could be shared through a sports committee-based approach:

“If HR are telling you what's been chosen, then others might not want to do that [sport]. But, if representatives are saying that they've made a joint decision across the company of what is going to be run for the year. They can get ideas from people about what they actually want” [P8: female team coordinator aged 27, NP].

Sharing the demands of organising and delivering team sport created a professional approach and a sense of control for the employer. This sense of control provided investment and support for an effective programme to be implemented, and therefore a facilitator to participation. In some cases, this input was delegated within the remit of human resources:

“It would have to have someone in HR maybe you'd appoint someone to be a manager or something. It would need careful running because otherwise it could very quickly fall apart” [P14: male head of public relations aged 40, NP].

#### 3.6.3. Funding

A lack of funding could demotivate participants from playing workplace team sport. However, it was the public sector organisations sampled in this study that were most frequently challenged by financial austerity and public accountability. For example, participation in an activity outside the traditional working practises may be perceived negatively by staff facing redundancy or presented unfavourably within the media:

“Money is a real challenge, and it's probably not the same for many organisations you talk too but also that it's public money. You've got to have a strong belief to say we're all going to go off and spend public money to play 5-a-side football. This is public money, and if you're going to spend £1000 on a sports hall for a year, so staff can go and play 5-a-side football, that's £1000 that's could have been spent on whatever. So, we do have to be accountable, we were concerned with what messages this will give out in the media” [P17: male workplace health advisor aged 40, NP].

Therefore, participation in self-funded informal groups could facilitate participation when funding was unavailable:

“So with football, we pay ourselves. It's only £4, it's not a big expense. We get the balls and equipment. People are comfortable paying for it” [P24: male technical leader aged 35, TS].

#### 3.6.4. Communication

Effective strategies that raised awareness and facilitated participation included a variety of visual (e.g., notice boards) and digital (e.g., staff intranet; social media) methods. A frequent communication method mentioned was virtual spaces (e.g., “Yammer”, social media and digital message boards). These virtual spaces enabled participation due to the level of flexibility, personal interaction and two-way communication they offered. In contrast, forms of communication without this two-way communication were reported to demotivate participation and create obstacles due to a lack of flexibility and availability offsite. For example, the intranet presented these obstacles to participation:

“It's a one-way portal, so unlike with an email where you can reply to it, you can't reply to it on the intranet. We put something out to the entire workforce. We are just pushing the message out there. There's no dialogue, no conversation there, you can't have a discussion about something or anything like that, so it doesn't work well for organising events. You can get two colleagues sat next to each other reading a thing about a softball match, and they'll be no action out of it because there's no discussion there” [P19: male senior corporate communication manger aged 27, TS].

#### 3.6.5. Workplace Culture

The culture within the workplace predisposed the adoption of workplace team sport. Culture was influenced by organisational determinants (i.e., practises; attitudes; behaviours), social norms (i.e., acceptance; understanding; support) and beliefs surrounding physical activity. For example, participants described how a culture of acceptance created support, encouragement and a facilitator for workplace team sport (e.g., *“You should embrace the company values of staying healthy and feeling fit” [P24: male technical leader aged 35, TS]*). Adopting the health promoting beliefs of the organisation led some participants to play team sport. Within a positive workplace culture, flexible working and the notion of “quality work over the quantity of work” was encouraged. Frequently, the importance of reinforcing flexible working was discussed with long-term participation in workplace team sport. Reinforcing flexible working led participants to perceive they had the freedom to take breaks during the working day to participate in activities such as team sport: *“They're fantastic here, they're all about flexible working [P23: male IT analyst aged 34, NP]”*. Likewise, within a positive workplace culture flexible working was frequently promoted and supported by supervisors: *“It's about output, rather than sitting at your desk, you have to manage people according to their needs [P7: female human resources manager aged 35, TS]”*.

A workplace culture encouraging flexible working provided trust, reinforcement and support for employees to take time out of the working day to participate in workplace team sport. Further, participants described how it was their employer's role to establish such a culture and their superior's role to reinforce this culture within the organisation:

“It comes back to my point of the manager setting the culture of an organisation. You know if the culture is that people work hard when they're at their desks, but they're allowed to get up from their desks and you know move about the office and take part in activities” [P21: female development manager aged 33, TS].

Alternatively, workplace culture created obstacles for participation in team sport. While, workplace team sport was not discouraged in any of the organisations sampled, a culture that encouraged “working none stop” was described:

“I don't think that I anticipate that I will likely to be participating. When I'm at work, I'm there to work. I just get as much done as possible then I can get home” [P4: female academic aged 48, NP].

Within this culture, participation in team sport was an additional recreational activity and therefore outside the remit of the working day. Moreover, an obstacle was created as finishing work before playing team sport was frequently reinforced through social norms:

“There is always that expectation that you do your work first, it is kind of an unsaid rule here” [P9: female solicitor aged 34, TS].

Likewise, a workplace culture that encouraged working non-stop was often reinforced by the attitudes and behaviour of superiors:

“If your manager turns up at eight and goes home a six, and never has a break. That's going to dictate the culture of your team to a degree. You've got a lot of pressure around that” [P17, male workplace health advisor aged 40, NP].

### 3.7. Environmental Factors Influencing Participation in Workplace Team Sport

#### 3.7.1. Sporting and Changing Facilities

The availability and quality of sporting and changing facilities either motivated or demotivated participants from playing workplace team sport. A lack of facilities within the workplace created a key obstacle for employees considering participation:

“It's actually physically doing it in the building or around the building. That is ultimately our biggest barrier” [P17: male workplace health advisor aged 40, NP].

Moreover, inaccessible facilities (e.g., *“I've got to get over there, I've got to do this and that, it's too long [P25]”*); health and safety protocols (e.g., *“the mountain of health and safety requirements, you would have to go through would be a nightmare [P4]”*); and unhygienic facilities created obstacles to participation (e.g., *“I know we have gyms, but they're a bit minging and I know the showers are crap [P19, TS]”*).

Alternatively, a lack of sports facilities may be overcome by utilising the accessible green space surrounding the workplace:

“Yeah so we're quite lucky. Where we are based there is a massive country park with a massive cricket ground there, which we have free reign over. The rugby club is round the back, we have access to their fields, there's a tennis court as well. So, on a summers night you just step out the back of HQ and you're there” [P19: male senior corporate communication manger aged 27, TS].

However, during the winter months these spaces became an obstacle to participation:

“Asking non-sports people to come out in the pouring rain and freezing cold to go play netball outside is going to be quite unlikely” [P1: female, aged 34, TS].

#### 3.7.2. The Support of External Sporting Organisations

Finally, some of the organisations sampled had a relationship with an external sporting organisation such as a national governing body, regional sports partnership or local sports club. These networks enabled individual's to deliver team sport within their workplace. Often, financial constraints and a lack of resources led organisations to seek support from external sporting organisations. External sporting organisations offer support by proving resources, sports leaders and education to deliver team sport:

“So we worked with four governing bodies that brought someone in to deliver it on the day. That person was a coach, because we recognised that not all people would have played the sports before. So, they organised it on the night, and delivered a short coaching session before” [P18: male sports development manager aged 41, TS].

### 3.8. Societal Factors Influencing Participation in Workplace Team Sport

#### 3.8.1. Bias and Inequality in Sport

Bias shaped by policy and teaching practises challenged some participants, and created a negative attitude towards participation in team sport. Negative experiences of “physical education” (PE), and more specifically with the style in which PE was delivered reduced perceptions of competence, self-efficacy and satisfaction with in sport in adult life:

“I think an experience of school sports, has put me off sport. At school, I had asthma, and when I was young when you had asthma you didn't do sports or only for a little bit in the summer so I was also the youngest in my year by quite some way. So, I was rubbish at sports, the rare times I did any and sports are all competitive, you did more if your good at it” [P4: female academic aged 48, NP].

Further, in some cases workplace champions created inequality in the delivery of workplace team sport by stereotyping the age and gender within their organisation:

“Well if we look at the demographic of what our employees' are, and we are a heavily female organisation, something in the region of eighty odd percentage are female and our average age is around the high thirty mark, say forty for the sake of argument. So, that in itself is a bit of a barrier” [P17: male, aged 40, NP].

Experiences of inequality perhaps explain why females in this study described intrapersonal obstacles such as a lack of perceived competence and reduced self-efficacy:

“I think it's more of a confidence thing. I don't think I would necessarily go, yeah I'll play football, as I'm terrible and I think for a woman you wouldn't necessarily feel confident doing it with work” [P10: female, aged 34, NP].

## 4. Discussion

This study used semi-structured interviews with employees to understand the complexity of participating in workplace team sport. Template analysis, interpreted through an ecological model, revealed participation in workplace team sport is influenced by (i) intrapersonal, (ii) interpersonal, (iii) organisational, (iv) environmental and (v) societal factors. More specifically, intrapersonal, interpersonal, organisational and environmental factors facilitate participation and create obstacles for employees considering or participating in workplace team sport. Further, factors shaped by societal attitudes such as bias and inequality in sport only challenged participation.

### 4.1. The Influence of Intrapersonal Factors

The findings of this study indicate intrapersonal factors can influence participation. Self-Determination Theory (SDT) proposes that supporting needs for autonomy, competence and relatedness can promote wellbeing and regulate autonomous motivation, while thwarting needs leads to illbeing and controlled forms of motivation [47]. Displays of competence and exercising in a group setting are associated with thwarted needs for competence and controlled introjected forms of motivation [47]. Therefore, it is unsurprising to note that antecedents of thwarted competence and introjected motivation such as diminished perceptions of competence, a negative experience of school sport, challenges surrounding body image and peer expectations created obstacles to participation in workplace team sport.

Further a fear of failure may reduce participation in workplace team sport [48]. Fears surrounding failure are created by social comparisons with a colleagues' performance in team sport or their colleagues' expectations of their performance in sport [38]. Likewise, unhealthy competitive behaviours (e.g., aggression, over-competitiveness, critiquing) were reported to reduce self-efficacy and thwart needs for competence and relatedness. Evidence, suggests self-efficacy and thwarted needs for competence and relatedness are associated with maladaptive forms of controlled motivation [49]–[51]. While, a fear of failure and the associated negative social comparisons are connected to reduced perceived competence, which likewise promotes the adoption of maladaptive behaviours (e.g., embarrassment; lack of self-efficacy; anxiety) [47],[48],[52],[53]. Further, such factors are known to underpin the adoption of controlled introjected forms of motivation and behaviour [47]. Controlled forms of motivation are known to predispose inconsistent participation and adherence [49]–[51].

Tailoring workplace team sport to support needs for competence, may promote more autonomous motivation and maintained behaviour. The findings of this study suggest competence can be supported through adapted or novel team sports. Sports with adapted rules or sports which are new to most may improve perceptions of competence and self-efficacy [53]. Evidence indicates this process is achieved through supporting and addressing challenges surrounding competence and facilitating antecedents of autonomous motivation, such as intrinsic motivation, enjoyment, and mastery experiences [49].

Participation and adherence to physical activity programmes is associated with supported needs for autonomy [49]–[51]. In this study, employees who participated in workplace team sport reported their enjoyment to predispose autonomous motivation. Supporting autonomy fosters psychological functioning, autonomous motivation and maintained behaviour [47],[49],[50]. Evidence suggests the adoption of more autonomous forms of motivation and behaviour can be achieved through the adoption of autonomy supportive leadership or coaching [49],[51]. Autonomy support provides an individual with a meaningful choice, reason and foundation for participation [49]. Within the workplace, autonomy support could be provided by champions who acknowledge the perspective of individuals and minimise the presence of pressure [50],[51],[53]. Researchers may consider adopting autonomy support strategies by encouraging workplace champions to adapt the rules of sport and imparting the benefits of team sport to employees [47],[49]–[51].

### 4.2. The Influence of Interpersonal Factors

In this study, interpersonal factors influenced participants contemplating or playing workplace team sport. The social relationships within workplace team sport reduced intrapersonal obstacles to participation, while commitments made to colleagues positively supported needs for relatedness [50],[51]. Further, the emotional support offered in a team environment may reduce obstacles surrounding a lack of self-efficacy and perceived competence [35],[54]. Qualitative evidence suggests supporting these markers of relatedness promotes more autonomously regulated forms of motivation [50].

The findings of this study suggest participants engaging in workplace team sport were motivated by determinants of relatedness such as group cohesion, identity and membership [47]. Evidence has suggested autonomous motivation and long-term exercise adherence are associated with supported needs for relatedness [49]. Further, determinants of relatedness such as cohesion and belonging to a group are known to be effective during the uptake and adherence to an activity [50]–[51]. The findings of this study add to evidence that suggests relatedness may fill a void prior to the point of complete autonomous motivation and maintenance behaviour [50],[51],[55]. Therefore, researchers may consider accounting for the presence and impact of relatedness when designing and implementing future programmes.

Consistent with recent evidence, balancing work and personal life was an obstacle for participants contemplating participation in workplace team sport [56]–[58]. Parents reported considerable challenges balancing participation in team sport and their role as an employee. Evidence exploring these challenges has indicated parents attribute their participation in physical activity to feelings of guilt and responsibility [35],[57]. The findings of this study however indicate offering team sport across a range of time-points, through autonomy-supportive participation (e.g., flexibility to attend) and accounting of the individual challenges balanced by their employees may improve participation and adherence. Therefore, researchers implementing team sport may wish to tailor not only to the needs of the organisation, but moreover to the personal demands that individual employees negotiate.

### 4.3. The Influence of Organisational Factors

The structure and culture of an organisation influenced the resources assigned to team sport, which therefore created a facilitator or obstacle for participation. Acceptance and support directly from colleagues and line managers, and indirectly from workplace champions and employers positively influenced participation and adherence in team sport. Evidence suggests employees seek understanding, acceptance and support from their colleagues, superiors and employer due to the demands shared by the workforce (e.g., job expectations, workloads) [59]. Further, the findings of the current study reinforce recent evidence exploring the experiences of employees participating in workplace physical activity schemes (i.e., some of which used team sports to promote health) [37]–[39]. These qualitative evaluations suggest a culture of acceptance and support meets needs for relatedness, provides wellbeing and self-efficacy and positively influences participation in team sport [37]–[39].

Employers were perceived by participants in this study to support workplace team sport through a duty of care for the workforce. Discussions within the literature suggest a duty of care is adopted due to the time employees spend in the workplace, pressure from government policy or health recommendations from external health promotion partners [18],[60]. Further, in the current study an indirect facilitator to workplace team sport may have been created when the duty of care extended to funding, communicating and managing workplace team sport programmes [60]. Therefore, researchers and practitioners may consider a participatory approach to workplace team sport interventions and programmes [61]. Adopting a participatory approach may secure support for the intervention (i.e., preparation phase), allow the researcher to address specific enablers and barriers (i.e., screening phase), develop the intervention around the necessary support (i.e., action planning phase) and implement the intervention with the required support (i.e., implementation phase) [61]. Further, a participatory approach provides researchers an opportunity to appraise and learn from the interventions implementation and effectiveness [61],[62].

Likewise, an employer's willingness to fund, communicate and support workplace health promotion enables a workplace champion or an occupational health team's ability to set-up, manage and deliver team sport [7],[36],[39],[56]. The findings of the current study suggest the presence of these factors can motivate participation in workplace team sport.

Within the organisations sampled, colleagues and superiors did not always directly support team sport. This lack of emotional, network, informational and tangible support might be explained from several organisational, interpersonal and intrapersonal standpoints. Evidence suggests the quantity of work is valued by the employer above the health of employees [59]. Likewise, evidence has suggested as workload increased acceptance for workplace team sport decreased [35]. The findings of this study indicate employees who participated in team sport were perceived as “not working” by their colleagues, while employees who “worked non-stop” were seen to be productive employees.

In the current study a workplace culture that encouraged participation in team sport, believed in the benefits of physical activity and promoted flexible working. A positive workplace culture is created through the emotional, esteem and network support of colleagues, line managers and employers, and may positively impact on organisational factors such as how team sport is perceived, communicated, funded and managed within a workplace [63],[64]. A workplace culture supportive of workplace team sport may influence the motivation, participation and adherence of the workforce through the social support and relatedness provided [35],[63],[64]. Further, a workplace culture supportive of team sport could be recognised as a method to promote interventions, schemes and programmes, due to its presence within the workplace and influence on individuals in a position of authority [36]. Further, occupational health teams, researchers and practitioners should consider the importance, complexity and influence of the workplace culture during the design and implementation of future health promotion programmes.

### 4.4. The Influence of Environmental Factors

Sporting and changing facilities predisposed participation in workplace team sport. In this study, inaccessible facilities challenged participation, while attainable facilities enabled participation. Empirical evidence has also demonstrated participation to be positively influenced by the provision of such facilities [25]–[28]. A prospective cohort study with Finnish public sector workers found an increased distance to sports facilities challenged participation and reduced physical activity levels, while a shorter attainable distance enabled participation and increased physical activity levels [19]. The current study expands on this evidence by suggesting sports and changing facilities are deemed inaccessible if they are unprofessional, unattainable or challenge the health and safety policy of the organisation. Likewise, evidence has suggested the provision of sports and changing facilities are determined by the level of funding and support within the workplace [65]. The findings of this study indicate the attitudes and decisions of employers may influence the funding available for sports facilities, resources and equipment [66]. Evidence using observational designs suggests employers are guided by beliefs of the benefits of participation, external inputs (e.g., government health promotion policy) and the perceptions and attitudes of their employees [39],[67]. While this study was unable collect data from employers, it appears that attitudes shape the perceptions and opinions of key decision makers such as senior leadership team members and managers. If workplace team sport programmes are to be successful and remain sustainable researchers and practitioners must influence the attitudes of employers through raising awareness of the benefits of team sport and influencing the creation of a workplace culture which is supportive of flexible working and physical activity [66],[67].

Further, self-presentation to managers, colleagues and clients remained important to participants, and frequently created an obstacle for participants playing and considering participating in workplace team sport. The findings of this study suggest limited changing facilities and the commute to the sports site combined with the attitudes of colleagues and line managers created an additional time based obstacle to participation, where employees were challenged by the time taken to return to work.

Social support theory proposes that an individual's wellbeing and self-efficacy for an activity (e.g., team sport) may be supported through emotional, esteem, network, information and tangible support [54]. For workplace team sport programmes to be successfully implemented the working practises and behaviours of employees and line managers should be addressed to provide emotional and network support for participation [54].

The findings of this study indicate, obstacles surrounding sports and changing facilities may be overcome by creating acceptance for extended breaks within the culture of the workplace and exploiting the environment surrounding the organisation (e.g., leisure centres, sports complexes or outdoor spaces) [36]. Further, creating a “novel event” such as team sport whereby the environment surrounding the organisation is utilised, may provide relatedness to employees willing to participate due to the social support and cohesion associated [50]–[51]. It is plausible participation may be improved as relatedness is associated with fostered wellbeing and the adoption of autonomous motivation [49],[55].

Further, the findings of this study indicate external sporting organisations (e.g., sports governing bodies, sports partnerships, clubs) may provide tangible and informational support for an organisation promoting workplace team sport [35],[54]. The findings of the current study suggest these external sporting organisations provide equipment, resources and knowledge to an organisation, and therefore employers may wish to create networks with these external sporting organisations when implementing workplace team sport.

### 4.5. The Influence of Societal Factors

Participants who chose not to participate in workplace team sport indicated that a negative experience of school sport reduced their perceptions of competence and self-efficacy. The findings of this study support evidence from prospective cohort designs which found the delivery, structure and content of PE influences adult participation in physical activity and sport [68]. Further the findings of this study indicate that the traditional delivery of PE valued performance outcomes (e.g., winning and performance) over the apparent health benefits [68]. This pedagogical tradition driven by education policy and societal attitudes is known to increase intrapersonal obstacles such as reduced self-efficacy and perceptions of a lack of competence [68]. Further, the findings of this study support evidence that suggests, these perceptions negatively influence the current physical activity behaviours of working age adults considering participation in workplace team sport [68],[69].

Therefore, it remains important for research to retrospectively understand the extent to which policy and teaching practise shape experiences of school sport and likewise participation in adult life. Researchers and practitioners using team sport to promote the health of the workforce should consider designing interventions, schemes and programmes that are underpinned by motivation theories such as SDT where an emphasis is placed upon the social environments' influence on competence, relatedness and autonomy [47]. SDT research has suggested perceptions of sport can be positively influenced by the adoption of more autonomy supportive coaching styles [49],[53].

Further, perceptions of competence and self-efficacy may be reduced by the inequality driven attitudes of individuals organising and delivering workplace team sport. The current study found, that some workplace champions, although not all, believed that women within their workplace do not enjoy team sports. However, it remains interesting to note that workplace champions reported these attitudes, rather than the six female participants who took part in workplace team sport themselves. While there were no reports of a “masculine” culture within the workplaces sampled, it should be noted that most the participants were male (i.e., 62.5% of team sport participants were male) despite the organisations sampled being a relatively equal split of genders. Participation levels and reports from champions highlight more serious questions of how team sport is promoted to female employees and if inequality exists in workplace health promotion.

Additional organisational obstacles may be created whereby due to perceptions of the demographic of the organisation by decision makers, team sport may be “overlooked” and unsupported by the organisation. Therefore, further investigation is required to understand the extent these attitudes shape the sports provisions offered within the workplace. Despite this lack of clarity, these attitudes go a way to explain why female participants in this study reported a lack of self-efficacy and made negative social comparisons surrounding performance with their peer group [70],[71]. Therefore, time should be invested in exploring the needs of female employees, and creating re-education strategies for occupational health teams and workplace champions that make workplace team sport appealing to women.

### 4.6. Methodological Considerations

Research exploring workplace team sport has been limited by homogenous samples [24]. This study attempted to addresses this limitation by purposively sampling participants from a range of industries within the UK. While, the sample in this study contained a diverse range of participants unavoidable their perceptions, ideas and opinions may not be representative of all employees. Further, most of these participants were sampled from large organisations. Therefore, the findings of this study mostly relate to large organisations, a point which should be considered when generalizing the findings. Future research, may wish to explore the facilitators and obstacles faced by employees in smaller organisations. Due to the size of the workforce, and structure and culture of these organisations, it is plausible additional facilitators and obstacles may emerge or be more prominent within the data. Moreover, the data collected in this study lacks the opinions, attitudes and perceptions of employers. On reflection, future research may wish to explore the opinions and attitudes of employers, and should consider this limitation when interpreting the findings of this study.

Likewise, no inactive participants volunteered to take part in this study. Moreover, the participants in this study considered themselves to be physically active and some of the samples were employed as health professionals within the promotion of sport and health. It is likely employees with an interest in sport or working within health promotion may face different barriers to inactive employees and may have a vested interest in participation [35]. For example, given the importance of competence and self-efficacy, it is plausible an employee who has not participated in team sport for a substantial period of time may face or perceived there to be greater challenges regarding the adoption of sports specific skills (e.g., passing the ball in soccer) than an employee who is already active or promoting sport within their job role. Furthermore, a workplace involved in the promotion of sport may provide social support for participation in a more meaningful way than a largely inactive workplace. Likewise, an environment and culture promoting participation in team sport may have consistent reminders to remain be active and participate in physical activity and therefore participation in team sport may be more socially acceptable than in a workplace which is largely inactive. Therefore, future research should consider exploring the barriers inactive employees face when negotiating participation in workplace team sport and examining the physical activity behaviour of employees participating in workplace team sport.

## 5. Conclusions and Implications for Future Research and Practice

This study explored the complexity of participating in workplace team sport. Qualitative research exploring the facilitators and obstacles faced by employees participating in workplace team sport is lacking and therefore challenging the implementation of programmes. Findings indicate participation in workplace team sport is influenced by interlinking intrapersonal, interpersonal, organisational, environmental and societal factors.

While the findings of this study share some similarities with previous evidence [34]–[39], an emphasis should be placed upon tailoring team sport to the needs and demands of the organisation and the employees which work within them. If team sport is to become a prevalent method of workplace health promotion, researchers must seek to influence the management, structure and culture of organisations. Accounting for these factors through case study designs may provide employees the support to participate in their workplace and additional insight into the challenges employers face when promoting team sport.

Further, the complexity of supporting an employee's experience of workplace team sport must be accounted for. Supporting basic needs for autonomy, competence and relatedness during workplace team sport is known to be associated with fostered wellbeing and autonomous motivation [49]–[51]. Therefore, researchers may wish to integrate the support basic needs into the designs of their interventions, schemes and programmes.

To understand if the findings of this study are consistent across a working age population, an intervention is required. A participatory approach would provide an understanding of the specific facilitators and obstacles encountered by participants and therefore allow researchers to tailor the intervention towards the necessary and required support [61],[62]. Whilst an intervention guided by SDT has the potential to train workplace champions in providing autonomy support, which is known to support basic needs [49]. The findings of the current study and past research support the use of this approach [49]–[51].

## Footnotes

1 Both of the researchers had experiences within an organisational setting. AB had previously managed within the retail industry, while JF undertook a placement in a human resources department within the manufacturing industry. AB is white British male aged 26, while JF is a white British female aged 22. By virtue of a lack of experience, both considered themselves naïve to the experiences faced throughout full-time employment and the impact of participating in workplace team sport.

## References

[1] World Health Organisation (WHO) (2015). Physical activity strategy for the WHO European region 2016–2025.

[2] Ekelund U, Steene-Johannessen J, Brown W (2016). Does physical activity attenuate, or even eliminate, the detrimental association of sitting time with mortality? A harmonised meta-analysis of data from more than 1 million men and women. Lancet.

[3] Hamilton M, Healy G, Dunstan D (2012). Too little exercise and too much sitting: Inactivity physiology and the need for new recommendations on sedentary behaviour. Curr Cardiovasc Risk Rep.

[4] Ding D, Lawson K, Kolbe-Alexander T (2016). The economic burden of physical inactivity: a global analysis of major non-communicable diseases. Lancet.

[5] Department of Health (DoH) (2011). Physical activity guidelines for adults (19-64 years).

[6] Townsend N, Wickramasighe K, Williams J (2015). Physical Activity Statistics 2015.

[7] Pronk N, Kottke T (2009). Physical activity promotion as a strategic corporate priority to improve worker health and business performance. Prev Med.

[8] Malik S, Blake H, Suggs L (2014). A systematic review of workplace health promotion interventions for increasing physical activity. Br J Health Psychol.

[9] Widera E, Chang A, Chen H (2010). Presenteesim: A public health hazard. J Gen Int Med.

[10] Amlani N, Munir F (2014). Does physical activity have an impact on sickness absence? A review. Sports Med.

[11] Evers K, Castle P, Prochaska J (2014). Examining relationships between multiple health risk behaviours, well-being, and productivity. Psychol Rep.

[12] Thorsteinsson E, Brown R, Richards C (2014). The relationship between work-stress, psychological stress and staff health and work outcomes in office workers. Psychol.

[13] del Pozo-Cruz B, Gusi N, Adsuar J (2013). Musculoskeletal fitness and health-related quality of life characteristics among sedentary office workers affected by sub-acute, non-specific low back pain: a cross-sectional study. Physiother.

[14] Lindwall M, Gerber M, Jonsdottir I (2014). The relationships of change in physical activity with change in depression, anxiety, and burnout: A longitudinal study of Swedish healthcare workers. Health Psychol.

[15] Dunstan D, Howard B, Healy G (2012). Too much sitting—a health hazard. Diabets Res Clin Pract.

[16] Public Health England (2014). Everybody Active, Every Day: An Evidence-Based Approach to Physical Activity.

[17] Black C, Frost D (2011). Health at work—An independent review of sickness absence.

[18] Batt M (2009). Physical activity interventions in the workplace: The rationale and future direction for workplace wellness. Br J Sports Med.

[19] Halonen J, Stenholm S, Kivimäki M (2015). Is change in availability of sports facilities associated with change in physical activity? A prospective cohort study. Prev Med.

[20] McEachan R, Lawton R, Jackson C (2008). Evidence, theory and context: using intervention mapping to develop a worksite physical activity intervention. BMC Public Health.

[21] Tavares L, Plotnikoff R (2008). Not enough time? Individual and environmental implications for workplace physical activity programming among women with and without young children. Health Care Women Int.

[22] Bennie J, Salmon J, Crawford D (2010). How do workplace environments influence physical activity? A qualitative study of employee's perceptions of influences on physical activity within the workplace. J Sci Med Sports.

[23] Veitch J, Clavisi O, Owen N (1999). Physical activity initiatives for male factory workers: gatekeepers' perceptions of potential motivators and barriers. Aust N Z J Public Health.

[24] Brinkley A, McDermott H, Munir F (2016). What benefits does team sport hold for the workplace? A systematic review. J Sports Sci.

[25] Barene S, Holtermann A, Oseland H (2016). Effects on muscle strength, maximal jump height, flexibility and postural sway after soccer and zumba exercise among female hospital employees: A 9-month randomised control trial. J Sports Sci.

[26] Barene S, Krustrup P, Holtermann A (2014b). Effects of the workplace health promotion activities soccer and zumba on muscle pain, work ability and perceived physical exertion among female hospital employees. PLoS ONE.

[27] Barene S, Krustrup P, Brekke O (2014a). Soccer and Zumba as health-promoting activities among female hospital employees: a 40-weeks cluster randomized intervention study. J Sports Sci.

[28] Barene S, Krustrup P, Jackman S (2013). Do Soccer and Zumba exercise improve fitness and indicators of health among female hospital employees? A 12-week RCT. Scand J Med Sci Sport.

[29] Joubert Y (2014a). Sports contribution to open communication in a workplace: a qualitative study. J Psychol Afr.

[30] Joubert Y, De Beer H (2010a). Experiences of employees who participate in organisational team sport activities. J Emerg Trend Econ Manag Sci.

[31] Joubert Y, De Beer H (2010b). Organisation team sport interventions to overcome diversity constraints in the workplace. Int J Divers Organ Communities.

[32] Joubert Y, De Beer J (2011). Benefits of team sport for organisations. South Afr J Res Sport Phys Educ Recreat.

[33] Joubert Y, De Beer J (2012). Organizational team sport as a diversity management intervention: A qualitative study. Afr J Bus Manag.

[34] Lee A, Diamant L (1991). Sports in the workplace do they pay?. Psychology of Sports, Exercise, and Fitness: Social and Personal Issues.

[35] Keegan R, Middleton G, Henderson H (2016). Auditing the socio-environmental determinants of motivation towards physical activity or sedentariness in work-aged adults: A qualitative study. BMC Public Health.

[36] Caperchionea C, Reida C, Sharp P (2015). How do management and non-management employees perceive workplace wellness programmes? A qualitative examination. Health Educ J.

[37] Carter A, Bishop D, Middleton G (2014). An evaluation of Lincolnshire Sports' ‘Workplace Challenge’ physical activity programme.

[38] Evans A, Carter A, Middleton G (2016). Personal goals, group performance and ‘social’ networks: Participants' negotiation of virtual and embodied relationships in the ‘Workplace Challenge’ physical activity programme. Qual Res Sport Exerc.

[39] Adams E, Twumasi R, Musson H (2016). County Sports Partnership Network Workplace Challenge Summary Evaluation Report.

[40] Sallis J, Owen N, Fisher E, Glanz K., Rimer B. K., Viswanath K. (2008). Ecological models of health behaviours. Health Behavior and Health Education: Theory, Research and Practise.

[41] McLeroy K, Bibeau D, Steckler A (1988). An ecological perspective on health promotion programs. Health Educ Behav.

[42] Golden S, Earp J (2012). Social ecological approaches to individuals and their contexts: twenty years of health promotion interventions. Health Educ Behav.

[43] Alvesson M, Ashcraft K, Symon G, Cassell C (2012). Interviews. Qualitative Organizational Research: Core Methods and Current Challenges.

[44] Hanna P (2012). Using internet technologies (such as Skype) as a research medium: A research note. Qual Res.

[45] Patton M (2002). Qualitative Research & Evaluation Methods.

[46] Brooks J, McCluskey S, Turley E (2015). The utility of template analysis in qualitative psychology research. Qual Res Psychol.

[47] Deci E, Ryan R (2000). Self-determination theory and the facilitation of intrinsic motivation, social development, and well-being. Am Psychol.

[48] Conroy D, Elliot A (2004). Fear of failure and achievement goals in sport: Addressing the issue of the chicken and the egg. Anxiety Stress Coping.

[49] Gucciardi D, Jackson B (2015). Understanding sport continuation: An integration of the theories of planned Behaviour and basic needs theory. J Sci Med Sport.

[50] Kinnafick F-E, Thøgersen-Ntoumani C, Duda J (2014). Physical activity adoption to adherence, lapse, and dropout: A self-determination perspective. Qual Health Res.

[51] Kinnafick F-E, Thøgersen-Ntoumani C, Duda J (2014). Sources of autonomy support, subjective vitality and physical activity behaviour associated with participation in a lunchtime walking intervention for physically inactive adults. Psychol Sport Exerc.

[52] Ntoumanis N, Thogersen-Ntoumani C, Smith A (2009). Achievement goals, self-handicapping, and performance: A 2 x 2 achievement goal perspective. J Sport Sci.

[53] Adie J, Duda J, Ntoumanis N (2008). Autonomy support, basic needs satisfaction and the optimal functioning of adult male and female sport participants: A test of basic needs theory. Motiv Emot.

[54] Schaefer C, Coyne J, Lazarus R (1981). The health-related functions of social support. J Behav Med.

[55] Markland D, Tobin V (2010). Need support and behavioural regulations for exercise among exercise referral scheme client: The mediating role of psychological needs satisfaction. Psychol Sport Exerc.

[56] Audrey S, Procter S (2015). Employers' views of promoting walking to work: a qualitative study. Inter J Behav Nutr Phys Act.

[57] Mailey E, Huberty J, Dinkel D (2014). Physical activity barriers and facilitators among working mothers and fathers. BMC Public Health.

[58] Fletcher G, Behrens T, Domina L (2008). Barriers and enabling factors for work-site physical activity programs: a qualitative examination. J Phys Act Health.

[59] Cole J, Tully M, Cupples M (2015). “They should stay at their desk until the work's done”: A qualitative study examining perceptions of sedentary behavior in a desk-based occupational setting. BMC Res Not.

[60] Robroek S, van de Vathorst S, Hilhorst M (2012). Moral issues in workplace health promotion. Int Arch Occup Environ Health.

[61] Nielsen K, Randall R, Holten A-L (2010). Conducting organisational-level occupational health interventions: What works?. Work Stress.

[62] Sørensen O, Holman D (2014). A participative intervention to improve employee well-being in knowledge work jobs: A mixed methods evaluation study. Work Stress.

[63] Cooper K, Barton G (2015). An exploration of physical activity and wellbeing in university employees. Perspect Public Health.

[64] Edmunds S, Stephenson D, Clow A (2013). The effects of a physical activity intervention on employees in small and medium enterprises: A mixed methods study. Work.

[65] van Bekkum J, Williams J, Morris P (2011). Cycle commuting and perceptions of barriers: stages of change, gender and occupation. Health Educ.

[66] Pescud M, Teal R, Shilton T (2015). Employers' views on promotion of workplace health and wellbeing: A qualitative study. BMC Public Health.

[67] Grossmeier J, Hudsmith N (2015). Exploring the valid proposition for workforce health: Business leader attitudes about the role of health as a driver of productivity and performance. Am J Health Promot.

[68] Kirk D (2005). Physical education, youth sport and lifelong participation: The importance of early learning experiences. Eur Phys Educ Rev.

[69] Bocarro J, Kanters M, Casper J (2008). School Physcial Education, Extracurricular Sport, and Lifelong Active Living. J Teach Phys Educ.

[70] Harry J (1995). Sport ideology, attitudes towards women, and anti-homosexual attitudes. Sex Roles.

[71] McGinnis L, McQuillan J, Chapple C (2005). I just want to play: Women, sexism, and persistence in golf. J Sport Soc Issue.

